# Reproductive Organ of Blow Fly, *Chrysomya megacephala* (Diptera: Calliphoridae): Ultrastructural of Testis

**DOI:** 10.1155/2011/690863

**Published:** 2011-08-07

**Authors:** Kabkaew L. Sukontason, Tarinee Chaiwong, Urai Chaisri, Hiromu Kurahashi, Michelle Sanford, Kom Sukontason

**Affiliations:** ^1^Department of Parasitology, Faculty of Medicine, Chiang Mai University, Chiang Mai, 50200, Thailand; ^2^College of Medicine and Public Health, Ubon Ratchathani University, Ubon Ratchathani 34190, Thailand; ^3^Department of Tropical Pathology, Faculty of Tropical Medicine, Mahidol University, Bangkok 10400, Thailand; ^4^Department of Medical Entomology, National Institute of Infectious Diseases, Tokyo 162 8640, Japan; ^5^Department of Entomology, Texas A&M University, TX 77845-2543, USA

## Abstract

This work presents the ultrastructure of testis of the medically important blow fly, *Chrysomya megacephala* (Fabricius) (Diptera: Calliphoridae) using light microscopy and electron microscopy. Reproductive organ of males was dissected to determine the testis in the pupal stage, 3-day-old flies and 7-day-old flies and observed under scanning electron microscopy (SEM) and transmission electron microscopy (TEM). SEM displayed a smooth surface which is occasionally penetrated by tracheoles. TEM of the testis in the pupal stage presents the thick testis wall covering underdeveloped cells containing a variable size of an electron-dense globule. For the 3-day-old males, the testicular wall is formed by an external layer, a peritoneal sheath, a muscular layer, a basement membrane, and a follicular epithelium. Follicular epithelium presented developing spermatozoa. Regarding the 7-day-old males, development of spermatozoa is apparent, displaying nucleus, centriolar adjunct, axoneme, and mitochondrial derivatives, with the 9 + 9 + 2 microtubule pattern of axoneme.

## 1. Introduction

*Chrysomya megacephala *(F.) is the medically important blow fly species in many parts of the world including Thailand. Larvae of this species have been reported in association with human corpses in several case situations [1]. Not only have their specimens been used to estimate the postmortem interval (PMI) in cases [2], but also to detect organophosphate poisoning in a putrefying body through larval analysis [3]. In Thailand, *C. megacephala* has been the primary species of fly found at death scenes involving exposed, burned, hanging, or floating corpses, in which the types of environment were quite varied, including forested, urban, and suburban areas [1].

The biology, particularly, the reproductive morphology of blow fly has been documented as the basic research in some species. Examples of this are provided by investigation under light microscopy in *Chrysomya pinguis, Chrysomya defixa *[4], *Calliphora pubescens, Calliphora testaceifacies *[5], and *Onesia accepta *[6]; while scanning electron microscopic (SEM) study has been employed for researches in *Lucilia cuprina *[7], *Cochliomyia hominivorax*, and *Cochliomyia macellaria *[8]; most of such works have involved the taxonomic identification. Regarding applied research, the research involving bacterium, *Rickettsiella *in the ovary of the Oriental cockroach, *Blatta orientalis *[9], insect virus Hz-2V in the reproductive organs of the female corn earworm moth, *Helicoverpa zea* (Lepidoptera), and infection of the endobacterium was exemplary [10]. Concerning such published information, it is the objective of this study to investigate the reproductive system of *C. megacephala*, focusing on the testis, using light microscopy (LM), scanning electron microscopy (SEM), and transmission electron microscopy (TEM). This information will establish baseline data on the reproductive system of this species. 

## 2. Materials and Methods

### 2.1. Fly Strains

The adult male *C. megacephala *used in this study was obtained from a laboratory colony located at the Department of Parasitology, Faculty of Medicine, Chiang Mai University, Chiang Mai (at 17–21°N, 98-99°E), Thailand. Laboratory colonies were maintained at a natural temperature of 24–28°C with a light/dark photoperiod of *≈*12 : 12 h. Larvae were fed a fresh pork liver diet. Adults were reared on two kinds of food: (1) a mixture of 10% (w/v) sugar solution and multivitamin syrup solution and (2) fresh pork liver (used as both a food source and oviposition site). 

### 2.2. Scanning Electron Microscopy (SEM)

The ultrastructure of testis was studied using SEM. Twenty, 3–5-day-old male flies, were sacrificed by placing them in a freezer set at 4°C for 15 minutes. The dead flies were dissected in normal saline under a dissecting microscope to obtain this organ. They were fixed in 2.5% glutaraldehyde in phosphate buffer (PB) at pH 7.4 at 4°C for 24 hours. After primary fixation, samples were rinsed with PB two times at 15 min intervals. The rinsed samples were then placed in 1% osmium tetroxide as a post-fixative at room temperature for 2-3 hours. Subsequently, they were rinsed twice with PB and dehydrated gradually at 30 minute intervals with increasing concentrations of ethanol 10%, 30%, 50%, 70%, 80%, 90%, 95% and twice with 100% ethanol, to replace water with ethanol. Following water displacement, the specimens were soaked in acetone twice for 30 minutes. Critical point drying (CPD) was performed. Specimens were then attached to aluminum stubs with double-stick tape and coated with gold (Au) in a sputter-coating apparatus before being viewed with a JEOL JSM-5910LV scanning electron microscope (JEOL: Japan). 

### 2.3. Transmission Electron Microscopy (TEM)

Testis dissected from the pupal stage (~4-5 day-old pupa), 3-day-old males and 7-day-old males, were processed for the TEM investigation. The procedures for dissection and fixation of organs for the TEM study were the same as previously described for the SEM study. After postfixation, the testis was cut into separate units using a razorblade. Subsequently, they were dehydrated and placed in acetone for 2 hours before being transferred to a solution of resin : acetone in a ratio of of 1 : 3 for 24 hours, 1 : 1 for 24 hours and 3 : 1 for 24 hours. Each specimen was then soaked in a solution of resin only for 3 hours twice. Specimens were then embedded in Spurr's resin by placing them into a plastic block and incubating at 70°C for 24 hours. A thick section (0.5 *μ*m) of each organ was cut with a glass knife on an Ultramicrotome (Becthai, USA). Ultrathin sections (90 nm) were prepared from these reembedded blocks, with serial sections collected from copper slot grids. Sections were poststained with uranyl acetate and lead citrate before examination by a Hitachi H700 transmission electron microscope (Japan) operated at 100 kV.

## 3. Results

**Figure 1:**
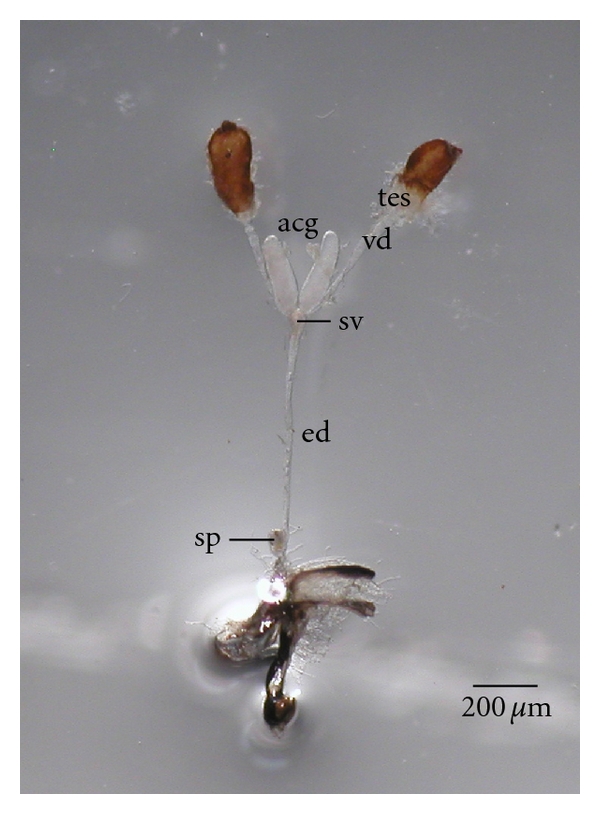
Whole internal reproductive tract in male *C. megacephala* showing paired testes (tes), paired vas deferens (vd), seminal vesicle (sv), paired accessory gland (acg), medial ejaculatory duct (ed), and sperm pump (sp).

**Figure 2:**
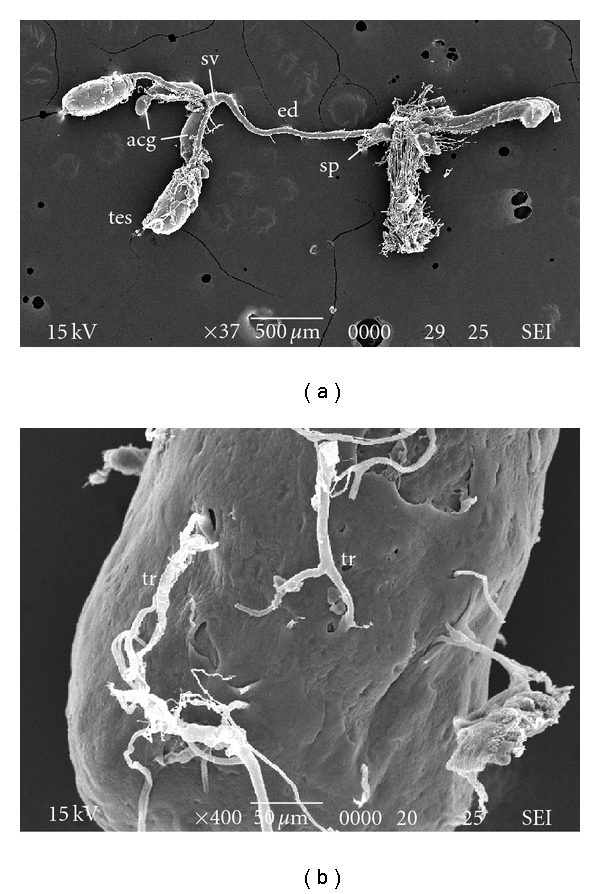
SEM image of internal reproductive tract in male *C. megacephala*. (a) Whole tract showing testes (tes), vas deferens (vd), seminal vesicle (sv), accessory gland (acg), ejaculatory duct (ed), and sperm pump (sp). (b) Testis showing the smooth surface and is occasionally penetrated by tracheoles (tr).

The internal reproductive organs in male *C. megacephala* comprised of pairs of testes, vas deferens and accessory glands, one seminal vesicle, one ejaculatory duct, and one sperm pump (Figures 1 and 2(a)). The testes were oval shaped, orange-brown in color, with the terminal end connecting to the vas deferens. The vas deferens was a thin, transparent, and long simple tube that opened into the seminal vesicle which is slightly enlarged and globular. The seminal vesicle is connected with the long, thin ejaculatory duct, of which terminal end was inserted into the sperm pump, connecting to the external genitalia.

Determination using light microscope revealed that the testes of *C. megacephala* are located in the posterior region of the abdomen and have an overall oval shape. The color of the testes of flies just after emergence is a pale orange. The color changes to become a reddish orange in 1-day-old flies. The color of the testes changes continuously from reddish orange (Figure  1(a)) to brown. 

**Figure 3:**
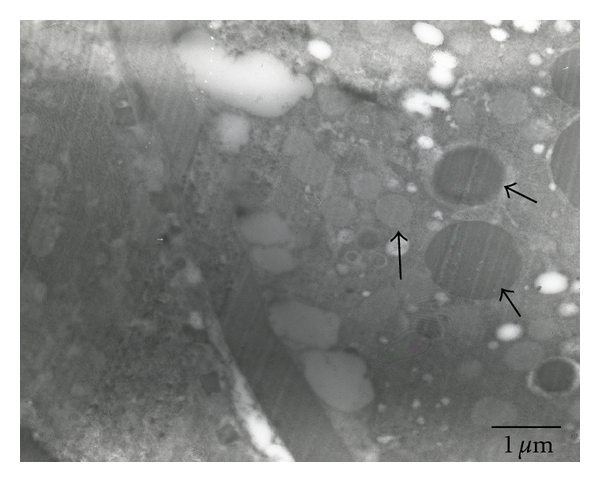
TEM micrograph of the testis of pupa *C. megacephala* showing thick testis wall covering underdeveloped germ cells with variable size and density of electron-dense globules (arrows).

**Figure 4:**
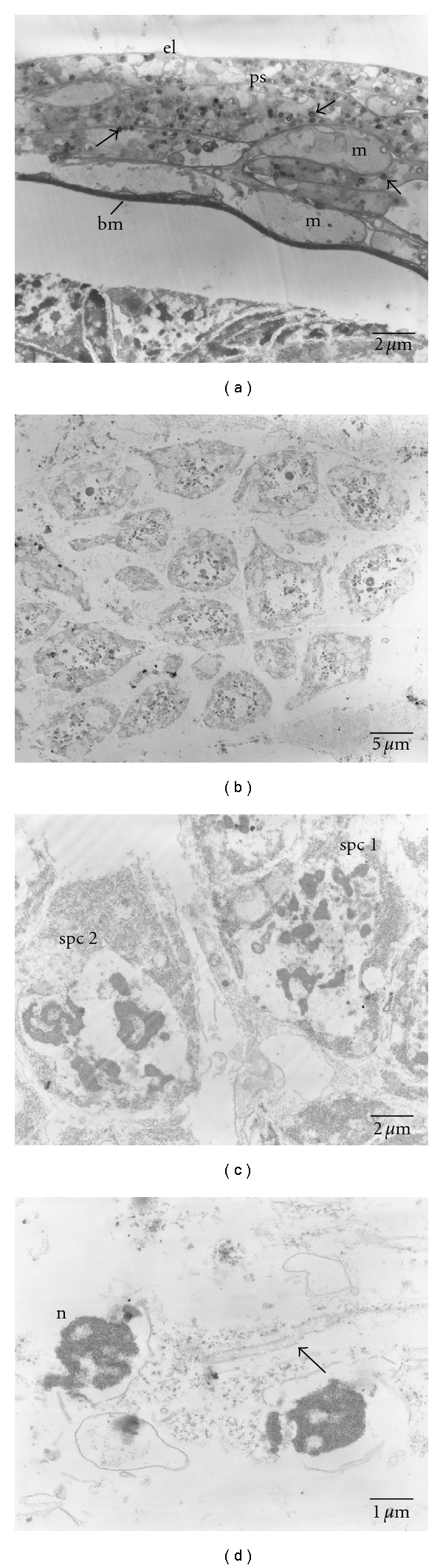
TEM micrographs of the testis of a 3-day-old *C. megacephala. *(a) Transverse section showing the strata forming the testicular wall formed by an external layer (el), a peritoneal sheath (ps), a muscular layer (m), and a basement membrane (bm). Rounded grains containing pigment, with varying density levels, are spread in the cytoplasm (arrows). (b) Germ cells showing group of pear-shaped cells. (c) Primary spermatocyte (spc 1) with packed electron-dense chromatin and secondary spermatocyte (spc 2) which is characterized by four groups of separated nuclei. (d) Spermatozoa with nuclei (n) and flagellum (arrow).

**Figure 5:**
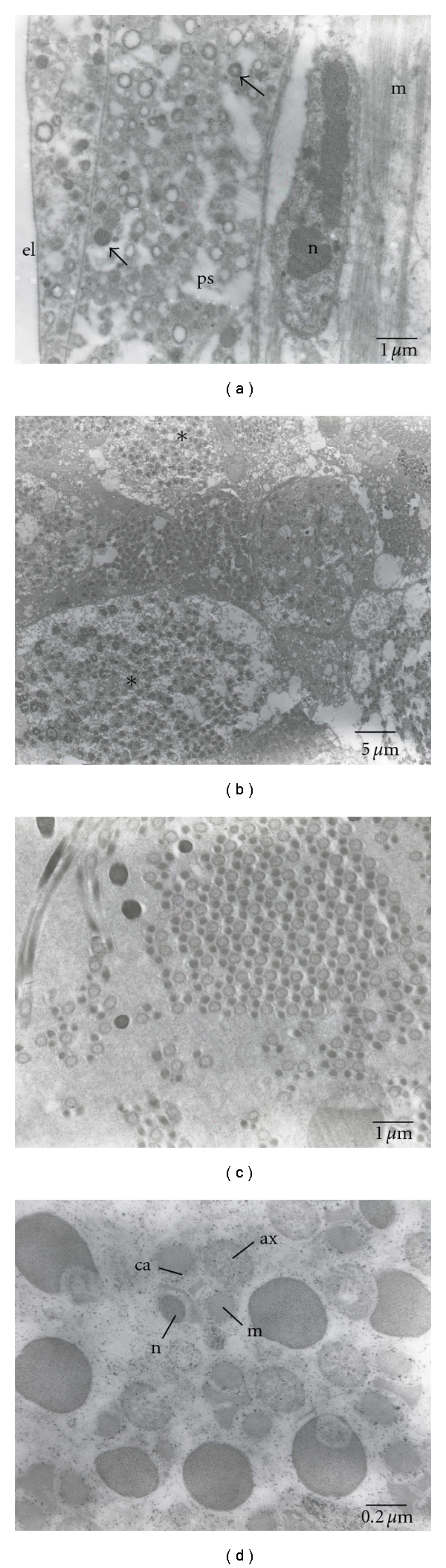
TEM micrographs of the testis of a 7-day-old *C. megacephala. *(a) Testicular wall displayed an external layer (el), a peritoneal sheath (ps), a muscular layer (m) with nucleus (n). Rounded grains containing pigment, with varying density levels, are spread in the cytoplasm (arrows). (b) Development of sperimatozoa inside the germ cells (asterisks). (c) Enlargement of (b) presenting group of spermatozoa. (d) Spermatozoa showing nucleus (n), centriolar adjunct (ca), axoneme (ax), and mitochondrial derivatives (m).

SEM micrographs of testes show a smooth surface that is occasionally penetrated by tracheoles (Figure 2(b)). Regarding TEM, micrographs of testes of pupal stage revealed the testicular wall covering underdeveloped cells containing a variable size and density of electron-dense globules (Figure 3). No apparent organelle was observed. For the 3-day-old males, the testicular wall is actually formed by an external layer, a peritoneal sheath, a muscular layer, and a basement membrane (Figure 4(a)). Rounded grains containing pigment, with varying density levels, are spread in the cytoplasm (Figure 4(a)). Observation at the follicular epithelium presented developing spermatozoa (Figure 4(b)). The first stage is the beginning of the first division of maturation, the primary spermatocyte. They are pear shaped with large nuclei. The ratio of nucleus and cytoplasm is 1 : 1, with chromatin that is dispersed inside the nucleus (Figure 4(c)). In the second division of maturation, the secondary spermatocyte is characterized by the presence of elongated clusters of chromatin in the globular-shaped cells. The primary and secondary spermatocytes can be differentiated in TEM micrographs (Figure 4(c)), where the third division of maturation is recognized by the appearance of spermatozoa with nuclei and flagellum (Figure 4(d)). Regarding the 7-day-old males, muscular layer is obvious in the testicular wall (Figure 5(a)). Development of spermatozoa inside testis is still observed (Figures 5(b) and 5(c)).  Figure 5(d) showed the composition of spermatozoa—nucleus, centriolar adjunct, axoneme, and mitochondrial derivatives.

## 4. Discussion

The internal male genital organs of *C. megacephala* consist of the various parts commonly found in insects. The testes are located in the posterior abdomen and contain all spermatozoa stages from primary spermatocytes to spermazoa. In almost all other Diptera, the testes are enveloped by two tissue layers consisting of the outer epithelium (or tunica externa) and the inner epithelium (or tunica interna). In species where color has been observed in the testes, pigment granules are deposited in the outer epithelium contributing to the apparent visible color [11]. As in other Diptera, the TEM micrographs of the testicular wall of *C. megacephala* show that the outer epithelium is full of the rounded grains containing the pigment, which give the organ its characteristic color. This characteristic has also been observed in the Mexican fruit fly, *Anastrepha ludens *[12]. 

SEM studies of the testes of various insects have shown both similarities and differences of *C. megacacephala*. The morphological characters of testes in four *Plecoptera* species consist of a number of separate follicles, each enclosed by its epithelium and open into the vas deferens [13]. In *Leuctra fusca*, each of the two testes consists of 9 to 10 follicles, almost tubular in shape; *Brachyptera risi*, the follicles are ovoid; *Taeniopteryx stankovichi*, the follicles are more roundish than those *B. risi*; *T. kuetreiberi,* the two testes are united in the median proximal portion, forming a single arc sac. In this current study, SEM investigations of male *C. megacephala* revealed a pair of oval-shaped testes with a smooth surface that is occasionally penetrated by tracheoles.

The presence of the primary and secondary spermatocytes observed in the developing spermatozoa of 3 day-old germ cells is similar to that observed in the pupal stage of the thrips, *Haplothrips simplex* (Thysanoptera: Phlaeothripidae) [14]. The tail region of *C. megacephala* spermatozoa as determined in 7-day-old males appeared likely consisting of two mitochondrial derivatives, an axoneme and an accessory body, of which 9 + 9 + 2 (9 outer single accessory tubule, 9 doublets and 2 central single) microtubule pattern of axoneme, and this has been recently clarified by Name et al. [15]. This pattern was also found in other blow fly, *Calliphora vomitoria *[16], flesh fly *Sarcophaga bullata* (Diptera: Sarcophagidae) [17]; fruit fly *Drosophila melanogaster* (Diptera: Drosophilinae) [18], *Orfelia* species, and *Boletina* species (Diptera: Mycetophilidae) [16] whereas in mosquito *Culex pipiens* and *Anopheles maculipennis* (Diptera: Culicidae) [16] and sand fly, *Phlebotomus papatasi* (Diptera: Psychodidae) [19] a type designated as 9 + 9 + 1. Nevertheless, the 9 + 9 + 0 type has been reported in other Diptera including the mosquito*, Toxorhynchites brevipalpis* (Diptera: Culicidae) [20].
